# Rheological and Injectability Evaluation of Sterilized Poloxamer-407-Based Hydrogels Containing Docetaxel-Loaded Lipid Nanoparticles

**DOI:** 10.3390/gels10050307

**Published:** 2024-05-01

**Authors:** Ana Camila Marques, Paulo C. Costa, Sérgia Velho, Maria Helena Amaral

**Affiliations:** 1 UCIBIO—Applied Molecular Biosciences Unit, MEDTECH, Laboratory of Pharmaceutical Technology, Department of Drug Sciences, Faculty of Pharmacy, University of Porto, 4050-313 Porto, Portugal; 2 Associate Laboratory i4HB, Institute for Health and Bioeconomy, Faculty of Pharmacy, University of Porto, 4050-313 Porto, Portugal; 3 i3S—Institute for Research and Innovation in Health, University of Porto, 4200-135 Porto, Portugal; 4 IPATIMUP—Institute of Molecular Pathology and Immunology of the University of Porto, 4200-135 Porto, Portugal

**Keywords:** intratumoral administration, injectable hydrogels, nanocomposite hydrogels, poloxamer, lipid nanoparticles, nanostructured lipid carriers (NLCs), docetaxel

## Abstract

Nanostructured lipid carriers (NLCs) have the potential to increase the bioavailability and reduce the side effects of docetaxel (DTX). However, only a small fraction of nanoparticles given intravenously can reach a solid tumor. In situ-forming gels combined with nanoparticles facilitate local administration and promote drug retention at the tumor site. Injectable hydrogels based on poloxamer 407 are excellent candidates for this hybrid nanoparticle–hydrogel system because of their thermoresponsive behavior and biocompatibility. Therefore, this work aimed to develop injectable poloxamer hydrogels containing NLCs for intratumoral delivery of DTX. To ensure sterility, the obtained hydrogels were autoclaved (121 °C for 15 min) after preparation. Then, the incorporation of NLCs into the poloxamer hydrogels and the impact of steam sterilization on the nanocomposite hydrogels were evaluated concerning sol–gel transition, injectability, and physicochemical stability. All formulations were extruded through the tested syringe–needle systems with acceptable force (2.2–13.4 N) and work (49.5–317.7 N·mm) of injection. Following steam sterilization, injection became easier in most cases, and the physicochemical properties of all hydrogels remained practically unchanged according to the spectroscopical and thermal analysis. The rheological evaluation revealed that the nanocomposite hydrogels were liquid at 25 °C and underwent rapid gelation at 37 °C. However, their sterilized counterparts gelled at 1–2 °C above body temperature, suggesting that the autoclaving conditions employed had rendered these nanocomposite hydrogels unsuitable for local drug delivery.

## 1. Introduction

Docetaxel (DTX) is a semisynthetic drug obtained by esterifying 10-deacetylbaccatin III, an inactive precursor molecule isolated from the needles of *Taxus baccata* [1,2]. As a member of the taxane family, DTX is an antimitotic agent used to treat a variety of cancers in both monotherapy and combination therapy. Nevertheless, there are significant limitations to the clinical application of DTX via intravenous administration, mainly its low solubility in water and systemic toxicity arising from its non-specific distribution in the body [3,4]. In addition to improving the pharmacokinetics and bioavailability of poorly water-soluble drugs, nanoparticle-based drug delivery systems may promote tumor-specific accumulation by passive or active targeting strategies due to their nanometric size and high surface area-to-volume ratio [5,6]. Lipid-based nanoparticles are a promising subtype, particularly the nanostructured lipid carriers (NLCs), which are primarily composed of solid and liquid lipids generally recognized as safe (GRAS) [7].

In a non-physiologically based analysis of 117 nanoparticle delivery studies published in 2005–2015, Wilhelm et al. [8] found that only 0.7% of the injected dose (ID) of nanoparticles reached the tumor. Using a physiologically based pharmacokinetic modeling approach [9], there was no significant improvement in the mean tumor delivery efficiencies estimated from data sets before 2015 (2.13%ID) and up to the year 2018 (2.33%ID). Very recently, Kumar et al. [10] obtained a median tumor distribution of 3.4%ID/g from a total of 2018 studies published up until April 2022. Considering that 0.7%ID is roughly around 3.2%ID/g, the median nanoparticle delivery to the tumor did not significantly improve between 2015 and 2022. Therefore, intratumoral administration should be chosen whenever it is feasible. Local therapies involving minimally invasive intratumoral injections provide a means of avoiding the obstacles that nanocarriers encounter in the bloodstream while sparing healthy tissues. As a result, there may be a higher accumulation of nanoparticles in the tumor and minimal off-target toxicity [11]. It is noteworthy, however, that drug-loaded nanoparticles injected directly into tumors tend to migrate away from the target site. To achieve the full potential of nanoparticles for local treatment, researchers have suggested combining them with hydrogels (HGs). Through depot formation and better retention within the tumor, the HGs can assist in localizing nanoparticles. The HG matrix can also modulate nanoparticle and drug release kinetics [12,13].

Injectable HGs have been developed to improve cancer diagnostics [14] and local treatment using chemotherapy [15] and immunotherapy [16,17]. Injectable HGs include pre-formed HGs with shear-thinning and self-healing properties and in situ-forming HGs [18]. In both cases, HGs are biodegradable and can be easily implanted into the body using a syringe or a catheter [19]. In situ-forming HGs are injected as free-flowing polymer solutions. Upon exposure to specific stimuli (e.g., light, enzymes, changes in temperature, pH, etc.), they transform into a non-flowing, gel-like depot at the injection site [20]. Unlike other stimuli-responsive HGs, thermoresponsive HGs relying only on a temperature stimulus can elicit the desired response without additional inputs, such as chemical initiators, enzymatic reactions, or equipment assistance. Not only is manipulation easier, but the manufacturing process is also less complex and more cost-effective [21,22].

Among thermoresponsive polymers, poloxamers have garnered attention in recent decades on account of their gelling behavior, affordability, and biocompatibility. Poloxamers are synthetic triblock copolymers consisting of poly(ethylene oxide) (PEO) and poly(ethylene oxide) (PPO) units (PEO-PPO-PEO). Given their amphiphilic character in aqueous solutions, these polymers self-assemble into micelles with a hydrophobic PPO core surrounded by a hydrophilic PEO shell [23]. Favoring the dehydration of PPO units, higher temperatures also initiate micelle formation, which marks the first stage of gelation. As the temperature rises beyond the critical solution temperature, micelles are rearranged into a cubic or hexagonal structure, leading to gelation [24].

With a molecular weight of approximately 12.6 kDa, poloxamer 407 (P407), also marketed as Pluronic^®^ F-127 (PEO_101_-PPO_56_-PEO_101_), is an FDA-approved excipient for pharmaceutical applications. The many advantages of P407, namely, its high solubilizing capacity, low toxicity, and minimal immunogenicity, have rendered it the material of choice for producing injectable HGs [25,26]. The literature has documented the use of nanocomposite HGs combining P407 HGs with various nanocarriers—polymeric nanoparticles [27], nanocrystals [28,29], hyaluronic acid-based nanocomplexes [30], and cyclodextrin inclusion complexes [31]—for intratumoral drug administration, which have demonstrated encouraging in vitro and in vivo outcomes for cancer chemotherapy.

Before being considered for clinical use, any material intended for close contact with the human body must be biocompatible and sterile [32]. Compared to other conventional methods for sterilizing HGs using ethylene oxide or gamma radiation, steam heat is a simpler, faster, and low-cost option that does not generate toxic waste. In steam sterilization, microorganisms are killed through irreversible protein denaturation caused by high temperatures and high humidity under pressure [33].

This work describes the development and characterization of injectable P407 HGs containing NLCs for intratumoral DTX delivery. The resulting nanocomposite HGs were evaluated in terms of sol–gel transition and injectability performance. Additionally, the influence of steam sterilization on their gelation, injectability, and physicochemical stability was also investigated.

## 2. Results and Discussion

### 2.1. Preparation of Nanocomposite Hydrogels

In this work, blank HGs and nanocomposite HGs incorporating unloaded NLCs (HG-NLC) or DTX-loaded NLCs (0.5 mg DTX per g of HG-NLC-DTX) were prepared (three batches of each) according to the “cold” method [34]. The final concentration of poloxamer for all HGs was set at 15% (*w*/*w*) following preliminary research on the sol–gel transition behavior of P407 HGs (Supplementary Figure S1).

**Figure 1:**
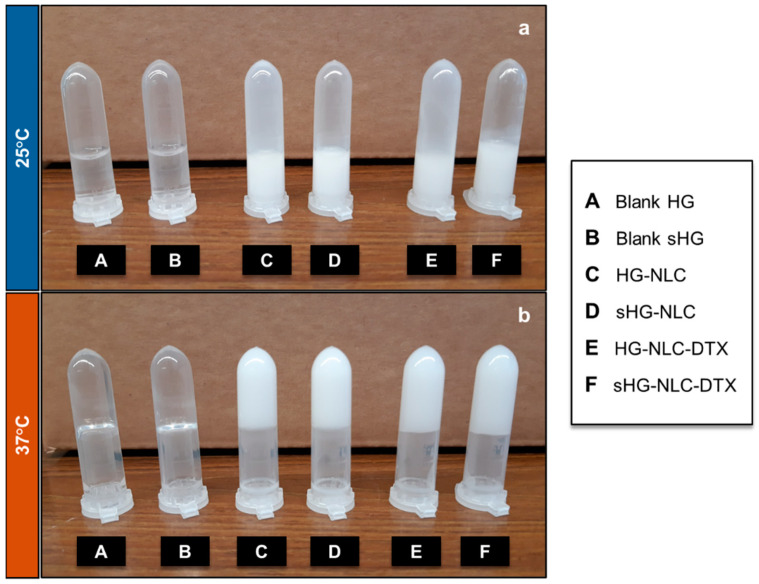
The aspect of sterilized (sHG) and non-sterilized (HG) blank and nanocomposite hydrogels—sol (**a**) and gel (**b**)—after incubation at 25 °C and 37 °C for a few minutes, respectively.

After preparation, blank HGs were transparent solutions at 4 °C, while nanocomposite HGs appeared as homogenous, low-viscosity, milky-white formulations due to the NLC dispersions. The nanocomposite HGs containing the DTX-loaded NLC (NLC-DTX) had the same post-production aspect as those containing the empty NLC. Macroscopic examination revealed no changes in their color or overall appearance after sterilization. Additionally, all HGs, whether sterilized or not, behaved as liquids (sol state) at room temperature (RT) and lost their ability to flow (gel state) at body temperature (Figure 1).

### 2.2. Characterization of Nanocomposite Hydrogels

#### 2.2.1. Rheological Behavior

Once the linear viscoelastic region (LVER) had been defined (Supplementary Figure S2), oscillatory tests were conducted to assess the sol–gel transition behavior (i.e., gelation) of the developed HGs. The impact of NLC incorporation and steam sterilization on gelation was investigated concurrently. The gel state and its stability at body temperature (37 °C) were also studied as a function of frequency.

The viscoelastic data obtained from the oscillatory rheological study include the storage (elastic) modulus (G′) and the loss (or viscous) modulus (G″). The former details the stored elastic energy, and the latter the energy loss caused by viscous deformation [35]. The phase angle δ (0° < δ < 90°) describing the relation between G′ and G″ components was also recorded [36].

##### Sol–Gel Transition

The intersection or crossover point of the moduli (G′ = G″ or δ = 45°) typically indicates the gel point, which was determined by running temperature and time sweep tests on the three batches of each HG.

The average curves for G′ and G″ as a function of temperature (4 to 50 °C), revealing three distinct regions, are depicted in Supplementary Figure S3. Both storage and loss moduli had low initial values, with the blank HG and all sHGs showing G″ dominance. Depending on the HG composition, G′ and G″ increased by different orders of magnitude in the second region, corresponding to the micellar phase [37]. The non-sterilized nanocomposite HGs (HG-NLC and HG-NLC-DTX) were “borderline” between the micellar and gel phases. Still, the prevalence of G″ at 25 °C for all formulations corroborated the viscous liquid (sol state) behavior at RT, as observed macroscopically (Figure 1). The thermally induced gelation was detected when the G′ values exceeded those of G″ (G′/G″ crossover). In the absence of NLCs, gelation is accompanied by a notable difference between the storage modulus and loss modulus values (G′~43.1 Pa vs. G″~16.7 Pa), particularly after sterilization (G′ of 1873 Pa vs. G″ of 774.3 Pa). The gel phase is the third region where elastic behavior prevails (G′ > G″).

**Figure 2:**
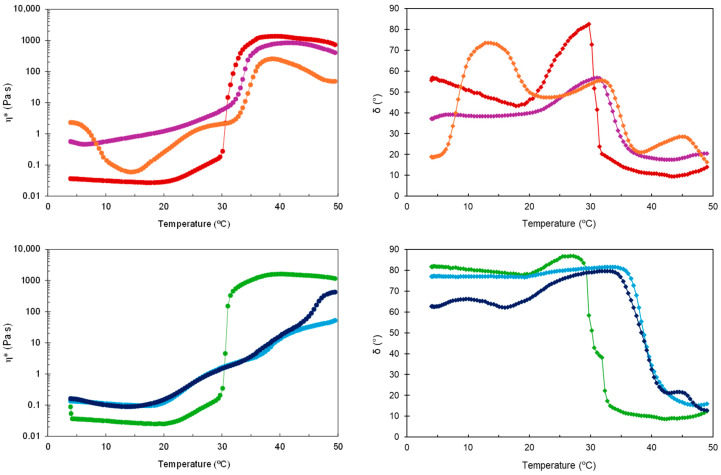
Complex viscosity (η*) and phase angle (δ) during temperature sweep for non-sterilized (**top**) and sterilized (**bottom**) HGs: blank HG (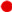,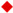), blank sHG (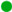,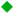), HG-NLC (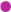,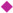), sHG-NLC (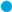,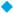), HG-NLC-DTX (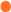,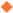), and sHG-NLC-DTX (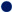,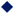).

Except for the two types of HG-NLC-DTX, the gel state of the other formulations was relatively stable up to 50 °C. While HG-NLC-DTX became less viscous around 39 °C, the sterilized counterpart experienced an increase in complex viscosity (η*) at temperatures above 45 °C (Figure 2).

Considering that G′ and G″ intersect at δ = 45° [38], the T_sol-gel_ values were calculated from the G′, G″, and δ curves vs. temperature through interpolation. Consistent with previous work [39,40], incorporating unloaded and DTX-loaded NLCs into P407 aqueous dispersions caused an increase in T_sol-gel_ from 30.8 °C to 33.1 and 34.3 °C, respectively. This suggests that the NLCs may interfere with micelle arrangement and packing, although the gelation temperatures obtained were still appropriate. For blank HGs, there was no change in gelation temperature after sterilization (T_sol-gel_ = 30.6 °C), contradicting previous observations of a slight reduction after autoclaving [41,42]. In the case of sterilized HG-NLC and HG-NLC-DTX, however, the solid-like behavior occurred above body temperature (39.0 and 38.4 °C, respectively), implying that the gelling ability could be lost in vivo.

**Figure 3:**
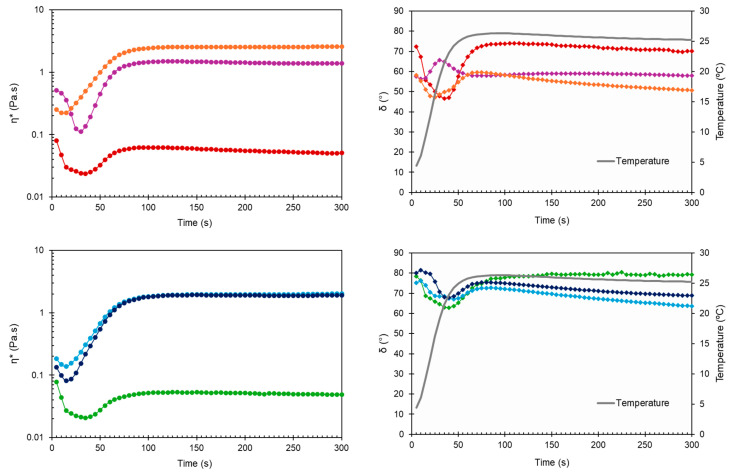
Complex viscosity (η*) and phase angle (δ) during time sweep at 25 °C for non-sterilized (**top**) and sterilized (**bottom**) HGs: blank HG (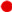,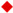), blank sHG (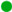,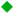), HG-NLC (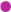,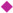), sHG-NLC (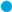,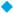), HG-NLC-DTX (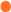,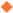), and sHG-NLC-DTX (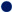,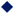).

**Figure 4:**
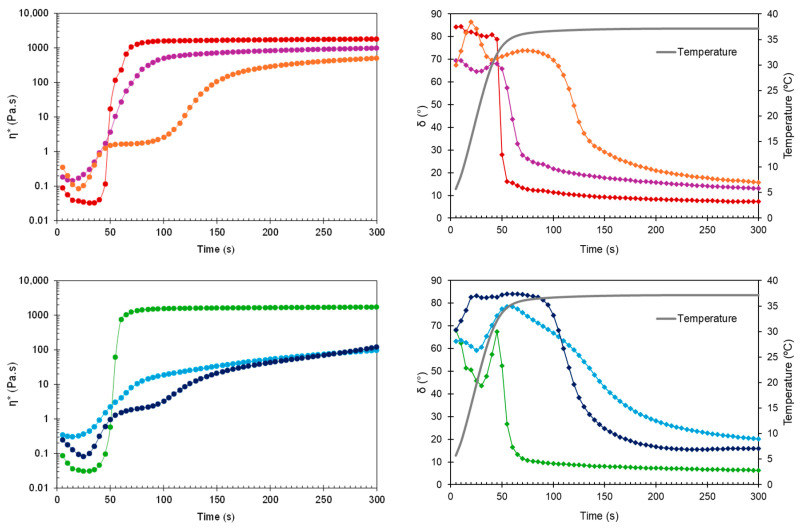
Complex viscosity (η*) and phase angle (δ) during time sweep at 37 °C for non-sterilized (**top**) and sterilized (**bottom**) HGs: blank HG (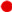,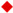), blank sHG (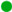,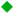), HG-NLC (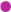,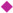), sHG-NLC (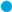,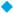), HG-NLC-DTX (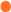,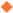), and sHG-NLC-DTX (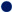,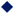).

The time-dependent changes in complex viscosity and phase angle at two relevant temperatures (25 °C and 37 °C) are presented in Figure 3 and Figure 4, respectively. With δ values always greater than 45°, the developed HGs behaved like liquids at RT, as required to facilitate injection.

Early in the time sweep test at 37 °C, all HGs were predominantly viscous (δ > 45°). However, the difference between physiological temperature and the obtained T_sol-gel_ values (30.6–33.1 °C) triggered a rapid phase transition, as evidenced by the drop in phase angle for blank HG, blank sHG, and HG-NLC below 45° (Figure 4, right). Based on Table 1 and Figure 4 (left), the presence of NLC-DTX delayed gelation and had a greater negative impact on viscosity than the empty NLC. Sterilization further decreased the G′ and G″ values of nanocomposite HGs and prolonged the gelation time of those with unloaded NLCs (70.7 ± 6.9 s). Regarding the complex viscosity (Figure 3 and Figure 4, left), the values were consistently less than 10 Pa·s at 25 °C. The viscosity values at 37 °C, on the other hand, were always greater than 10 Pa·s, occasionally surpassing 10,000 Pa·s. Compared to blank HGs, the viscosity of nanocomposite HGs was higher at 25 °C and lower at 37 °C. Whereas the increase in viscosity may be explained by the high concentration of nanoparticles in poloxamer dispersions, the reduction can be related to micelle–nanoparticle interactions during gelation.

To determine the gelation time, the time needed for the rheometer lower plate to reach the target temperature (37° C) was subtracted from the time required for G′ and G″ to cross (δ = 45°). A short gelation time reduces the risk of burst release due to drainage at the injection site and dilution by body fluids [43,44]. In line with the results of the temperature ramp test, both blank HGs and HG-NLC gelled before the plate reached 37 °C. Differently, HG-NLC-DTX only gelled in 50.8 ± 5 s. Interestingly, despite the gelation temperature of sterilized nanocomposite HGs, these formulations began to gel at 37 °C in a couple of minutes.

##### Frequency Sweeps

Tumor tissues can expand as they grow and undergo cell contraction as well [45]. Therefore, injectable HGs for local treatment should be evaluated for their ability to withstand tumor-related movements during growth and interaction with surrounding tissues.

**Figure 5:**
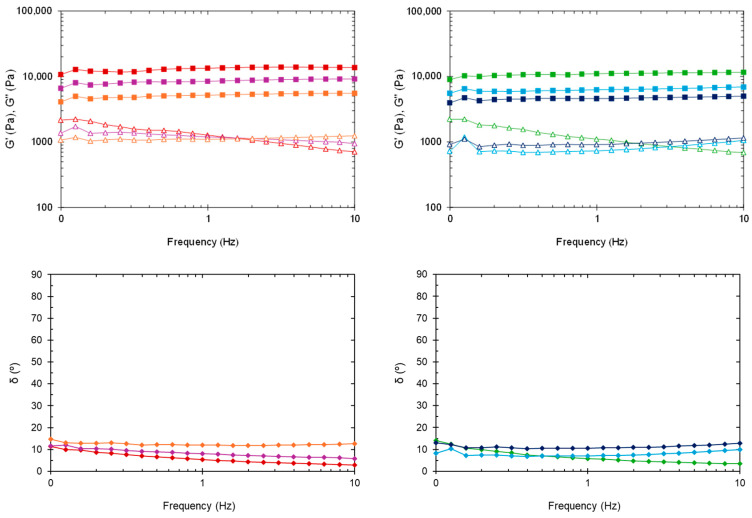
Oscillatory frequency sweep at 37 °C. Storage modulus (G′), loss modulus (G″), and phase angle (δ) of non-sterilized (**left**) and sterilized (**right**) HGs: blank HG (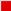,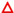,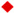), blank sHG (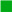,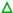,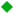), HG-NLC (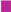,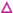,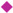), sHG-NLC (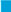,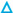,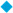), HG-NLC-DTX (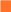,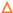,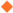), and sHG-NLC-DTX (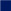,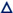,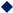).

Figure 5 shows the changes in dynamic moduli and phase angle at 37 °C in the frequency range from 0.1 to 10 Hz. The G′ value for all formulations was consistently higher than the G″ value, revealing a gel-like character and stability across the tested range. Overall, G′ and G″ were almost independent of frequency, though the loss modulus of blank HGs slightly decreased with increasing frequency. Even after sterilization, the viscoelastic behavior of nanocomposite HGs exhibited less frequency dependence than the blank HGs. The G′ and G″ values taken from frequency sweeps are in fair agreement with the data obtained from time sweeps at 37 °C (Table 1). One exception is sHG-NLC, which had a higher G′ than both HG-NLC-DTX and sHG-NLC-DTX.

In the plot of phase angle against frequency (Figure 5, bottom), the δ values (2.96–14.82°) were somewhat constant and below 45°, underlining the solid-like behavior of the formulations [46].

Collectively, these results suggest that none of the HGs developed would collapse or lead to burst release when subjected to tumor-associated movements [47].

#### 2.2.2. Injectability

In situ gelling systems for intratumoral administration require not only gelation under physiological conditions but also injectability, which refers to the force or work needed to expel the formulation from a syringe through a needle [48,49]. Although viscoelastic data sheds light on injectability performance, injection force establishes whether the formulation is appropriate for injection. The work of injection should also be evaluated to better characterize the extrusion of syringe content [50].

Injectability was quantitatively assessed at 25 °C using a texture analyzer in compression mode (5 kg load cell and 0.5 N trigger force) and a 2.5 mL syringe with 18-gauge (18G) or 21-gauge (21G) needles.

**Figure 6:**
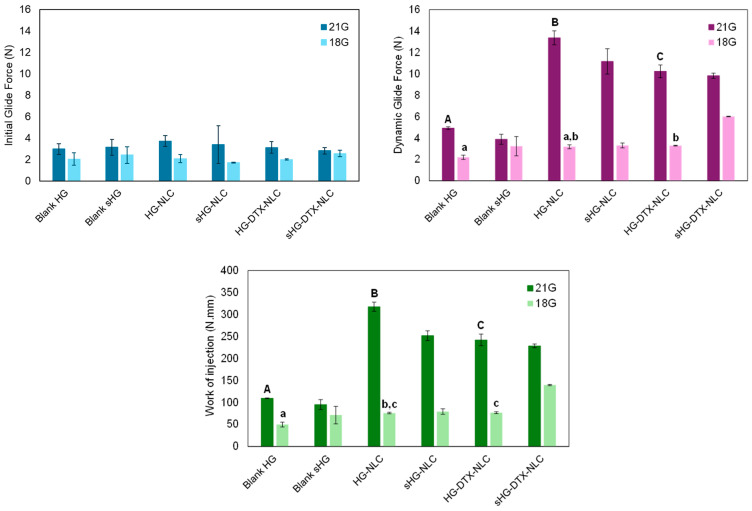
Initial glide force, dynamic glide force, and work required for injecting developed hydrogels through 18G and 21G needles. Error bars represent mean ± SD (n = 3). Data were analyzed using ANOVA followed by Tukey’s HSD post-hoc test, with different letters representing statistically significant differences (*p* < 0.05) between non-sterilized hydrogels injected through 18G (lowercase letters) and 21G (capital letters) needles.

In the force vs. distance plots of the developed HGs (Supplementary Figure S4), two distinct phases can be identified. The first event relates to the force necessary to move the plunger. The maximum force is followed by a plateau, after which the formulation is extruded through the needle with a relatively constant force. During this phase, the average force required to sustain the plunger movement is defined as the dynamic glide force [51]. The initial glide force (IGF), dynamic glide force (DGF), and work of injection (i.e., area under the curve) needed to extrude 2 mL of each HG were calculated from the recorded force vs. distance plots (Figure 6). The influence of the needle (18G or 21G) used, NLC incorporation into HG, and HG autoclaving on the extrusion of syringe content (injectability) was examined.

Apart from HG-NLC-DTX (*p* = 0.018), the IGF values of each HG injected with different needles did not differ statistically. The same was true for the IGF values of blank HGs in comparison to their nanocomposite counterparts before and after sterilization. This means that the force required to start the plunger movement was not dependent on HG type. Moreover, all formulations passed through 18G and 21G needles, with the DGF values ranging from 2.2 to 13.4 N. The gauge number, which is inversely related to the diameter of the needle [52], made a significant difference in the injection process, except for blank sHG. Particularly, the higher the gauge number, the greater the maximum force of injection: 6.0 ± 0.03 N for 18G vs. 13.4 ± 0.7 N for 21G. Still, the DGF values were far below the maximum force of 40 N that medical staff can apply during injection [53] and rendered the developed HGs clinically relevant (<20 N) [54]. Therefore, even the narrower gauge needle (21G) may be employed for these formulations to achieve a more precise injection [55]. It is worth noting that the obtained DGF values are likely lower than the actual values because tissue resistance during in vivo injection is disregarded [56]. As expected, changing the needle from 18G to 21G significantly increased the work of injection for blank HGs (*p* = 0.001) and all nanocomposite HGs (*p* < 0.001).

When using an 18G needle, the injection of HG-NLC-DTX resulted in DGF and area values comparable to those of HG-NLC but higher than those of blank HG (*p* = 0.049 and *p* = 0.024, respectively). The work of injection also increased with the addition of unloaded NLCs to poloxamer HGs (*p* = 0.031). With the incorporation of NLCs and the use of 21G needles, DGF and injection work were shifted to higher values (HG-NLC > HG-NLC-DTX > blank HG). It was surprising that HG-NLC, the least viscous nanocomposite HG at 25 °C (Figure 3, left), required the highest force and work of injection.

Few differences were observed in DGF and injection work between non-sterilized and sterilized HGs. Specifically, steam sterilization facilitated the administration of HGs with incorporated NLCs through a 21G needle by decreasing the DGF values from 13.4 ± 0.7 N to 11.2 ± 1.2 N (*p* = 0.012) and the area values from 317.7 ± 10.3 N·mm to 251.7 ± 11.3 N·mm (*p* < 0.001). Differently, autoclaving led to a rise in both parameters for sHG-NLC-DTX injected through the 18G needle. This unexpected effect of sterilization on force and work parameters contrasts with the viscosity of sHG-NLC-DTX at 25 °C (Figure 3, left), which was lower than that of the non-sterilized counterpart. This discrepancy can be attributed to increased local resistance caused by the needle’s diameter narrowing during fluid inflow and outflow [57]. Overall, the force used to inject the formulations through the 18G needle followed a similar pattern to the viscosity of HGs at RT.

#### 2.2.3. Chemical and Thermal Stability

##### Chemical Characterization

**Figure 7:**
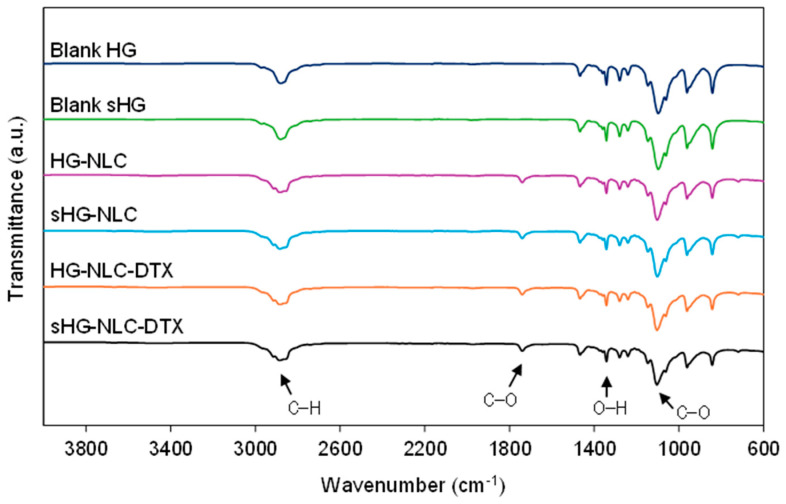
Fourier-transform infrared (FTIR) spectra of the sterilized (sHG) and non-sterilized (HG) blank and nanocomposite hydrogels. The graphs are plotted on the same scale to allow for easy comparison.

The developed HGs were examined by Fourier-transform infrared (FTIR) spectroscopy to detect changes in chemical structure resulting from steam sterilization. According to Figure 7, the molecular fingerprints of the freeze-dried HGs reflected the spectrum of P407. The characteristic absorption peaks of the polymer were found in all spectra at 2880 cm^−1^ (aliphatic C–H stretching), 1342 cm^−1^ (in-plane O–H bending), and 1100 cm^−1^ (C–O stretching) [58].

As for the nanocomposite HG spectra, the peak at about 1738 cm^−1^ (C–O stretching) was attributed to Precirol^®^ ATO 5, the primary lipid in the NLC composition. The other absorption peaks related to Precirol^®^ ATO 5 were found at 2914 and 2850 cm^−1^ (C–H stretching) and 1470 cm^−1^ (C=C stretching) in the NLC and NLC-DTX spectra (Supplementary Figure S5) [59,60]. In the presence of NLCs, the absorption band at 2880 cm^−1^ evolved into a shoulder arm due to C–H stretching in Precirol^®^ ATO [61]. No additional peaks were identified in the spectra of nanocomposite HGs containing DTX. Overall, the FTIR spectra revealed comparable intensities, with minor differences following NLC incorporation and after sterilization in the case of HGs with encapsulated DTX. Still, each HG had spectral overlap with its sterilized counterpart. These observations demonstrate that the surface functional groups and chemical bonding of the developed HGs were preserved during steam sterilization.

##### Thermal Properties

**Figure 8:**
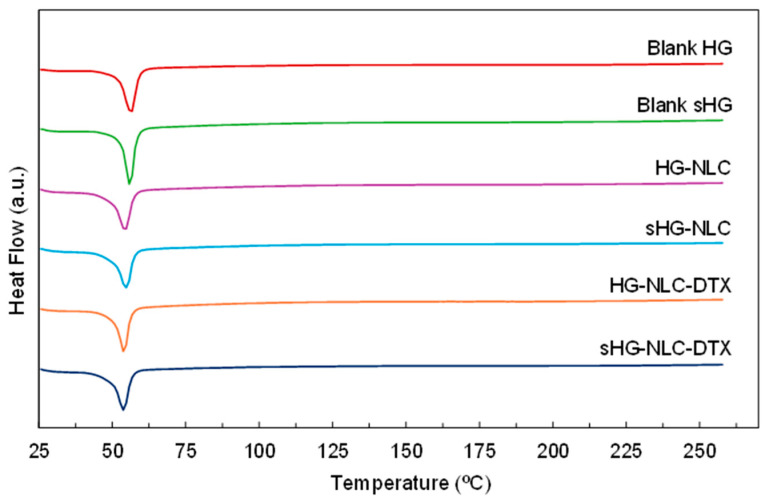
Differential scanning calorimetry (DSC) thermograms of the sterilized (sHG) and non-sterilized (HG) blank and nanocomposite hydrogels. The graphs are plotted on the same scale to allow for easy comparison.

Differential scanning calorimetry (DSC) provided complementary information on HG stability following sterilization in terms of thermal behavior. The results of the thermal analysis are presented in Figure 8 and Table 2.

All HGs exhibited an endothermic event corresponding to the melting point of P407 (Tm range: 52–57 °C) [62]. Although the peak location in the heating curves of nanocomposite HGs remained mostly constant, the onset and melting points calculated by the Proteus^®^ 8.0.1 software showed a slight shift toward lower temperatures. The incorporation of NLCs also translated into a reduction in melting enthalpy, referring to the integrated area under the peak [63]. This was slightly accentuated by autoclaving in the case of unloaded NLCs, as a tiny amount of poloxamer might have been degraded during the sterilization process. Such a hypothesis aligns with the increased gelation temperature (Table 1) of sHG-NLC due to a decrease in the effective polymer weight fraction [64].

However, steam sterilization and enthalpy values did not consistently correlate. The sterilizing treatment had the greatest impact on the blank sHG, increasing the enthalpy value by more than 30 J/g. Surprisingly, while the gelation and injection performance of the blank sHG changed very little, autoclaving appeared to have some effect on this thermophysical property.

## 3. Conclusions

To address the limitations of DTX systemic therapy, the authors developed and characterized an injectable nanocomposite poloxamer HG for intratumoral delivery of this drug. Since sterility is a requirement for considering the potential clinical use of any injectable system, the obtained HGs were sterilized using steam heat, and the effects of this process on their gelation, injectability, and physicochemical properties were studied.

The incorporation of the developed NLC-DTX into P407 dispersions produced nanocomposite HGs with the desired thermoresponsive behavior, as they behaved like low-viscosity fluids at 25 °C and rapidly gelled at temperatures near body temperature. Moreover, gel stability at 37 °C across a frequency range also pointed to their ability to resist tumor-related movements without collapsing or accelerating drug release. This also applies to the unloaded NLC, meaning the suggested HGs could also be useful for local injection of other hydrophobic drugs rather than DTX.

Although nanocomposite HGs required more effort to inject at 25 °C than the blank HGs, the work of injection was appropriate, and the obtained force values complied with the recommendations for clinical use. Overall, sterilized formulations were more easily injected than their non-sterilized counterparts. According to the FTIR and DSC results, the chemical structure and thermal properties of HGs appeared to be fairly well preserved during sterilization. However, this process raised the gelation temperature of nanocomposite HGs by 1–2 °C above body temperature, and thus, gelation in vivo cannot be guaranteed. This limitation could result in DTX being rapidly cleared from the tumor into the systemic circulation, negating the benefits of local administration. The impact of steam sterilization at 121 °C for 15 min on gelation is sufficient to conclude that these autoclaving conditions may not be suitable for the developed HGs.

As observed by Burak et al. [65], sterilization temperature is likely to be a crucial factor in this process. Therefore, future studies should consider autoclaving at a lower temperature for an extended period before rejecting the possibility that this sterilization method is appropriate for nanocomposite poloxamer HGs.

## 4. Materials and Methods

### 4.1. Materials

Stearic acid (C_18_H_36_O_2_; melting point: 69–70 °C), Miglyol^®^ 812 (medium-chain triglycerides), and Tween^®^ 80 (polysorbate 80) were purchased from Acofarma (Madrid, Spain). Precirol^®^ ATO 5 (glyceryl palmitostearate) was kindly provided by Gattefossé (Saint-Priest, France). Docetaxel, 99% (molecular weight: 807.88 g/mol), was purchased from Thermo Fisher Scientific Inc. (Waltham, MA, USA). Pluronic^®^ F-127 (molecular weight: ~12,600 g/mol) was purchased from Sigma-Aldrich (St. Louis, MO, USA). All water used (type 1 water) was obtained from a Milli-Q Direct-Q^®^ 3 UV-R Water Purification System (Merck KGaA, Darmstadt, Germany).

### 4.2. Preparation of Nanocomposite Hydrogels

**Figure 9:**
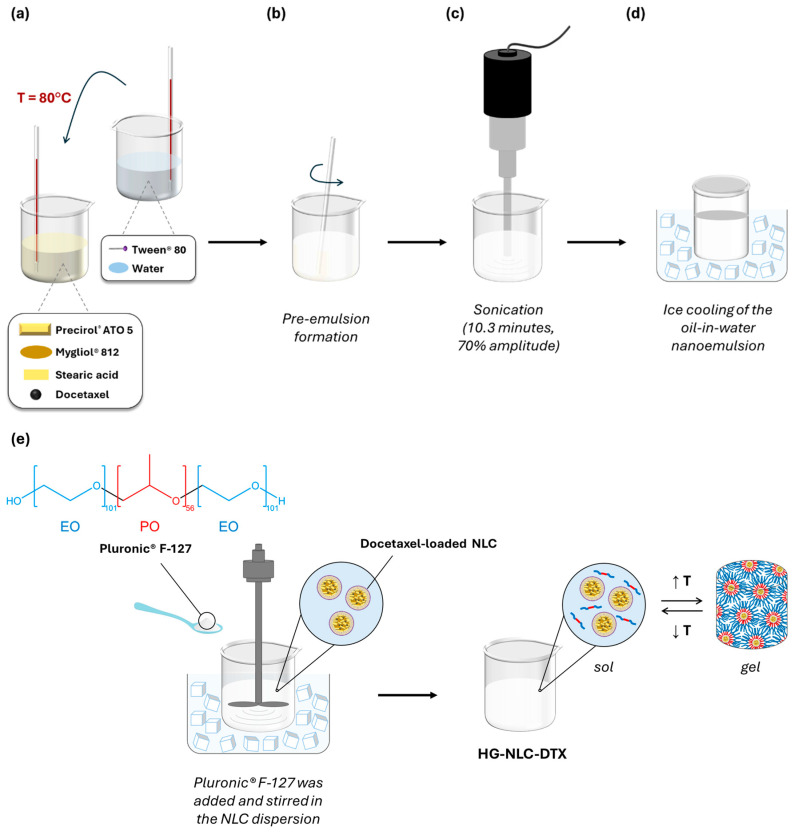
A schematic representation of the preparation process for Pluronic^®^ F-127 hydrogels containing a docetaxel-loaded NLC (HG-NLC-DTX): (**a**) the addition of the aqueous phase to the lipid phase after heating both to 80 °C; (**b**) the dispersion of the lipid mixture in the aqueous solution until a pre-emulsion is formed; (**c**) sonication; (**d**) rapid cooling of the nanoemulsion to obtain NLC-DTX; and (**e**) the preparation of the thermoresponsive nanocomposite hydrogel HG-NLC-DTX using the “cold” method.

First, NLC dispersions were produced by means of sonication, as depicted in Figure 9.

The lipids used in this formulation were Precirol^®^ ATO 5, Mygliol^®^ 812, and stearic acid, while the selected surfactant was Tween^®^ 80.

In brief, the lipid and aqueous phases were initially heated separately at approximately 80 °C, which is 10 °C above the melting point of stearic acid. The aqueous phase was then added to the molten lipid mixture (Figure 9a), which was dispersed in the aqueous solution containing Tween^®^ 80 as an emulsifier, forming a pre-emulsion (Figure 9b). Following this, sonication was applied for 10.3 min at a 70% amplitude, using a Sonics Vibra-Cell™ probe (CV18, Sonics & Materials Inc., Newtown, CT, USA) (Figure 9c). The resultant oil-in-water emulsion was transferred to a glass vial and rapidly cooled down for 20 min in an ice bath to form the NLC (Figure 9d). When preparing NLC-DTX, the drug was dissolved in the heated lipids before adding the aqueous phase.

To prepare blank HGs and nanocomposite HGs (HG-NLC and HG-NLC-DTX), an appropriate amount of P407 was dispersed in cold ultrapure water or NLC dispersion at 750 rpm for 1.5 h, using a mechanical stirrer (Heidolph RZR 2041, Heidolph Instruments GmbH & Co. KG, Schwabach, Germany) (Figure 9e). The sterilized counterparts were prepared as previously described, kept at 4 °C for 48 h, and then steam sterilized (121 °C for 15 min) in an autoclave (Uniclave 88, AJC, Cacém, Portugal). All HGs were stored under refrigeration (4 °C) [42].

### 4.3. Characterization of Nanocomposite Hydrogels

#### 4.3.1. Rheology

A week following the preparation and sterilization of the HGs, rheological measurements were conducted on a Kinexus lab+ rotational rheometer (Malvern Instruments Ltd., Worcestershire, UK) using a parallel plate measuring system with a 1.0 mm working gap. Oscillatory tests were performed at a constant strain within the LVER to preserve the HG microstructure against the deformation applied. The measured viscoelastic parameters were complex viscosity (η*), storage modulus (G′), loss modulus (G″), and phase angle (δ).

The LVER for the blank and nanocomposite HGs was initially identified by an amplitude sweep test (0.1–100%) at a frequency of 1 Hz, and the shear strain was then fixed at 0.15%. By running a temperature sweep from 4 to 50 °C at a heating rate of 5 °C/min, the gelation temperature was estimated from the G′/G″ crossover point, which corresponds to the gelation point [66]. To examine the gelling behavior of the HGs at both room and body temperature, as well as to determine gelation time, time sweeps of up to 5 min were carried out independently at 25 °C and 37 °C. For both temperature and time sweep tests, the lower plate was cooled to 4 °C before loading each HG between the plates, and frequency was maintained at 1 Hz. Finally, the viscoelastic properties and state of the developed formulations were also investigated with a dynamic frequency sweep (10–0.1 Hz) at 37 °C [67]. Rheological data were processed with the rSpace for Kinexus software (version 2.0.0.0, NETZSCH-Gerätebau GmbH, Selb, Germany).

#### 4.3.2. Injectability Test

**Figure 10:**
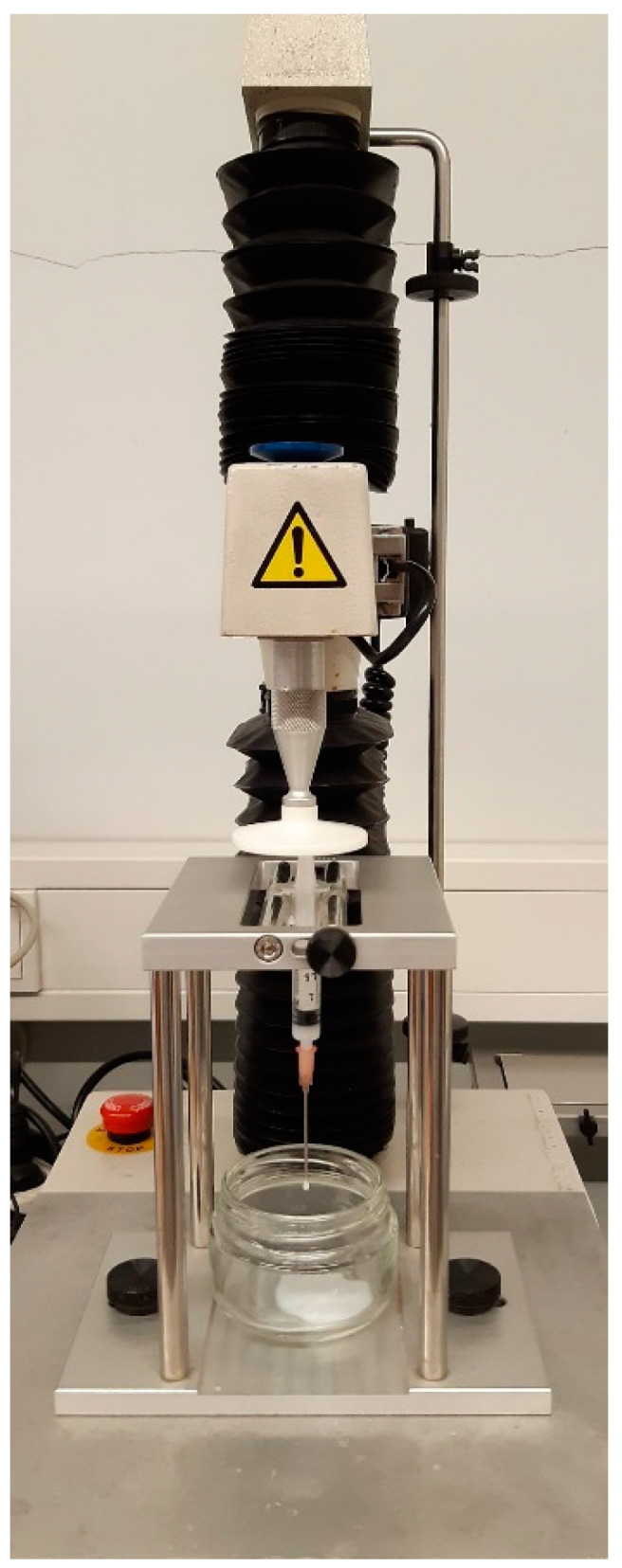
The experimental setup of the injectability test using a TA-XT2i texture analyzer.

A texture analyzer (TA-XT2i, Stable Micro Systems, Surrey, UK) with a 5 kg load cell was used to assess the injectability of both the sterilized and non-sterilized HGs. The HGs were left overnight at 25 °C before testing. A total of 2 mL of each HG was placed in 2.5 mL luer slip syringes (Pic Solution^®^) fitted with 18Gx1 1/2″ (1.20 × 40 mm) or 21Gx1 1/2″ (0.80 × 40 mm) needles. The syringe–needle system was fixed with a vertical holder, as shown in Figure 10. After the trigger force of 0.5 N was achieved, a compression plate located above the syringe plunger descended 30 mm at a velocity of 1 mm/s, simulating the typical speed of manual injection [53].

Some important parameters of injectability, such as initial glide force (IGF), dynamic glide force (DGF), and work of injection, were calculated from the recorded force–distance plots using the Texture Exponent 32 software (version 6.1.26.0, Stable Micro Systems, Surrey, UK).

**Figure 11:**
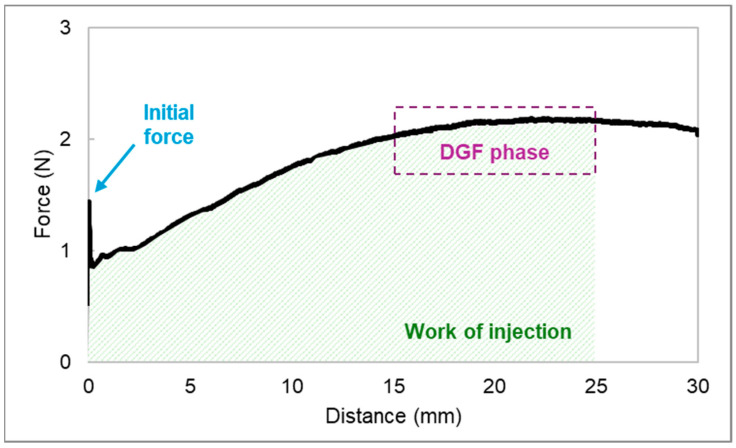
The force–distance profile of a 15% (*w*/*w*) P407 hydrogel in a 2.5 mL syringe extruded through an 18G needle. Three parameters related to injectability were assessed: initial glide force (IGF), dynamic glide force (DGF), and work of injection.

The force–distance profile of the blank HGs extruded through an 18G needle is displayed in Figure 11.

Whereas IGF is the force (N) required to initiate the plunger movement, DGF refers to the force needed to sustain the plunger movement during injection [68]. The latter is given by the mean force (N) of injection taken from the plateau of the curve, which is located between 15 and 25 mm. The work required to extrude the syringe content was correlated with the area under the curve (N·mm) [69]. Three injections “into the air” were performed for each HG and the results are presented as mean ± standard deviation (SD) (n = 3).

#### 4.3.3. Fourier-Transform Infrared Spectroscopy

FTIR spectroscopy was used to characterize the developed HGs regarding their chemical stability and integrity after sterilization [70]. The sterilized and non-sterilized HGs were frozen at −80 °C and lyophilized using a LyoQuest freeze dryer (Telstar, Terrassa, Spain). Both NLC and NLC-DTX were also tested after being lyophilized. Each sample was placed on a PerkinElmer Frontier™ FTIR spectrometer (Waltham, MA, USA) equipped with a diamond attenuated total reflectance system. The spectra were obtained by collecting 32 scans between 4000 and 600 cm^−1^ with a resolution of 8 cm^−1^, using the PerkinElmer Spectrum™ 10 software. The results presented for each HG are the average of two measurements.

#### 4.3.4. Differential Scanning Calorimetry

DSC measurements were conducted to evaluate whether the temperature applied during steam sterilization impacted the thermal properties of the HGs [71]. The DSC 214 Polyma^®^ equipped with an automatic sample changer (NETZCH-Gerätebau GmbH, Selb, Germany) was calibrated with pure indium for melting point and heat of fusion. The freeze-dried HGs (3.1–5.1 mg) were weighted into aluminum crucibles and hermetically sealed. An empty sealed crucible was used as the reference. All samples were scanned from 25 to 260 °C at a heating rate of 10 °C/min in a nitrogen atmosphere (flow rate: 50 mL/min) [72]. The data were analyzed using the Proteus^®^ software provided with the DSC equipment (version 8.0.1, NETZCH-Gerätebau GmbH, Selb, Germany).

#### 4.3.5. Statistical Analysis

The injectability data were analyzed using a one-way analysis of variance (ANOVA), followed by multiple comparisons with Tukey’s HSD test. *p*-values < 0.05 indicate statistical significance. The results of initial glide force, dynamic glide force, and work of injection are presented as the mean ± SD of three replicas. The statistical analysis was performed with IBM SPSS Statistics software for Windows (version 28.0, IBM Corp., Armonk, NY, USA).

## Figures and Tables

**Table 1:** Rheological characterization of developed hydrogels in terms of gelation temperature, gelation time, and viscoelastic data obtained from time sweeps at 1 Hz.

HG	T_sol-gel_ (°C)	Gelation Time (s)	25 °C	25 °C	37 °C	37 °C
			G′ (Pa) *	G″ (Pa) *	G′ (Pa) *	G″ (Pa) *
Blank HG	30.8 ± 0.6	0	0.11	0.30	11,216.7	1418.3
Blank sHG	30.6 ± 1.5	0	0.08	0.30	10,737.3	1137.7
HG-NLC	33.1 ± 0.6	0	4.63	7.33	6040.3	1106.0
sHG-NLC	39.0 ± 0.4	70.7 ± 6.9	5.81	11.24	546.3	255.8
HG-NLC-DTX	34.3 ± 0.5	50.8 ± 5.0	10.34	12.55	3036.7	824.8
sHG-NLC-DTX	38.4 ± 1.3	40.1 ± 6.5	4.17	11.11	700.5	268.5

Gelation temperature (T_sol-gel_) and gelation time are expressed as mean ± standard deviation (*n* = 3). * The storage modulus (G′) and loss modulus (G″) are the average values recorded at the end of each time sweep.

**Table 2:** Thermal properties of sterilized (sHG) and non-sterilized (HG) blank and nanocomposite hydrogels.

HG	Onset (°C)	Peak (°C)	Enthalpy (J/g)
Blank HG	52.7	56.8	150.9
Blank sHG	53.2	56.4	181.1
HG-NLC	50.5	54.2	144.0
sHG-NLC	49.9	53.4	134.1
HG-NLC-DTX	51.7	54.7	124.6
sHG-NLC-DTX	50.2	53.8	144.7

## Data Availability

The data supporting the reported results can be found in the article and the Supplementary Materials.

## Supplementary Materials

The following supporting information can be downloaded at: https://www.mdpi.com/article/10.3390/gels10050307/s1, Figure S1. Storage modulus (G′) and loss modulus (G″) during temperature sweep for P407 hydrogels at 14% (,), 15% (,), and 16% (,) (*w*/*w*); Figure S2. Amplitude sweep test at 37 °C and 1 Hz. Storage modulus (G′), loss modulus (G″), and phase angle (δ) for non-sterilized blank HG (,,) and HG-NLC (,,); Figure S3. Storage modulus (G′) and loss modulus (G″) during temperature sweep for blank HG (,), blank sHG (,), HG-NLC (,), sHG-NLC (,), HG-NLC-DTX (,), and sHG-NLC-DTX (,). Figure S4. The force–distance profiles of the developed hydrogels in a 2.5 mL syringe extruded through 18G (top) and 21G (bottom) needles. The curves represent the average results of three injections. Figure S5. Fourier-transform infrared (FTIR) spectra of the unloaded NLC and the DTX-loaded NLC (NLC-DTX). The graphs are plotted on the same scale to allow for easy comparison.
