# Supplementary material for: Rheological and Injectability Evaluation of Sterilized Poloxamer-407-Based Hydrogels Containing Docetaxel-Loaded Lipid Nanoparticles
Source: Gels. 2024 May 1;10(5):307. doi: 10.3390/gels10050307 (PMC11121564; doi:10.3390/gels10050307)
Supplement: Supplementary file 1 [file gels-10-00307-s001.zip › gels-2966753-supplementary.pdf]

Rheological and Injectability Evaluation of Sterilized Poloxamer-407-Based Hydrogels Containing Docetaxel-Loaded Lipid Nanoparticles

Ana Camila Marques [1,2,*], Paulo C. Costa [1,2], Sérgia Velho [3,4] and Maria Helena Amaral [1,2,*]

1 UCIBIO—Applied Molecular Biosciences Unit, MEDTECH, Laboratory of Pharmaceutical Technology, Department of Drug Sciences, Faculty of Pharmacy, University of Porto, 4050-313 Porto, Portugal
2 Associate Laboratory i4HB, Institute for Health and Bioeconomy, Faculty of Pharmacy, University of Porto, 4050-313 Porto, Portugal
3 i3S—Institute for Research and Innovation in Health, University of Porto, 4200-135 Porto, Portugal
4 IPATIMUP—Institute of Molecular Pathology and Immunology of the University of Porto, 4200-135 Porto, Portugal
* Correspondence: amarques@ff.up.pt (A.C.M.); hamaral@ff.up.pt (M.H.A.)

Supplementary Materials

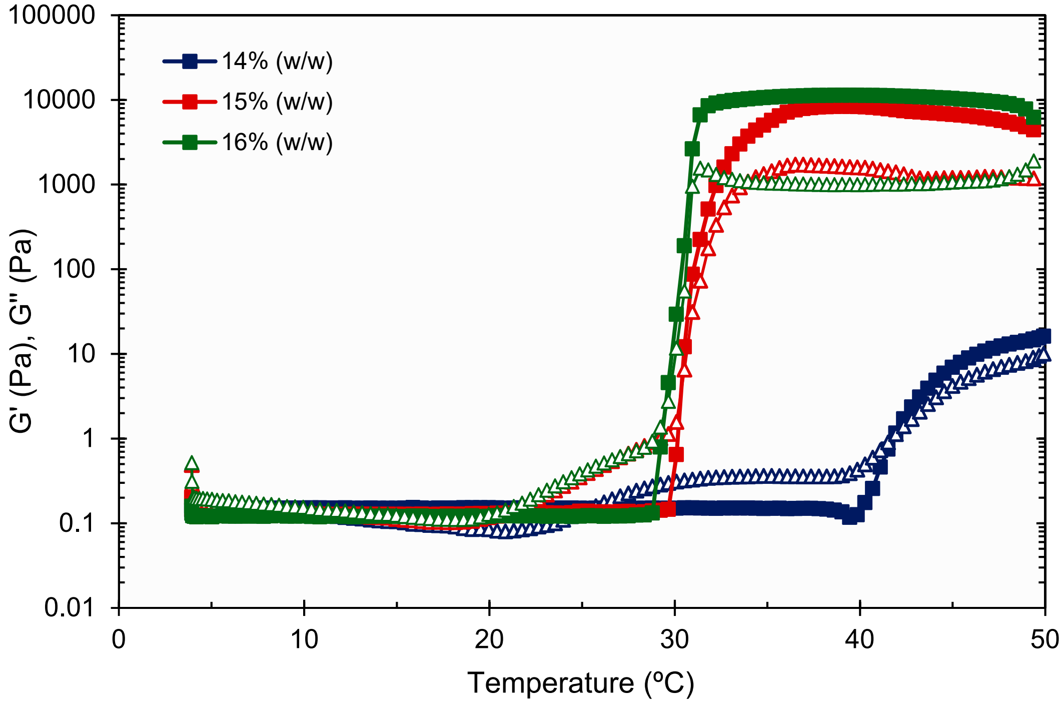

Figure S1. Storage modulus (G') and loss modulus (G'') during the temperature sweep for P407 hydrogels at 14% (■ , △), 15% (■ , △), and 16% (■ , △) (w/w).

The concentration of poloxamer 407 (P407) was chosen according to gelation temperature. To be appropriate for in vivo applications, gelation temperature should be close to body temperature but never higher than 37°C. As expected, poloxamer concentration and gelation temperature varied inversely. Gelation temperatures for P407 hydrogels at 14%, 15%, and 16% (w/w) were 41.8 ± 0.5, 30.8 ± 0.6, and 29.6 ± 0.1°C, respectively.

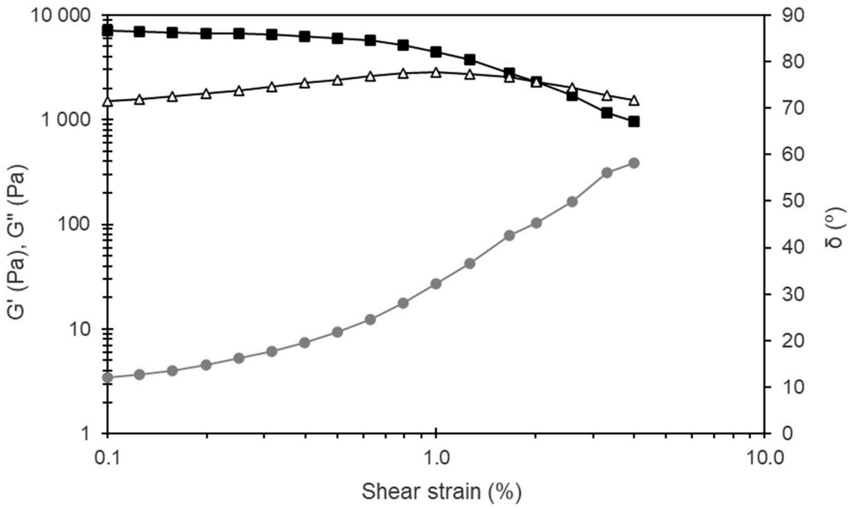

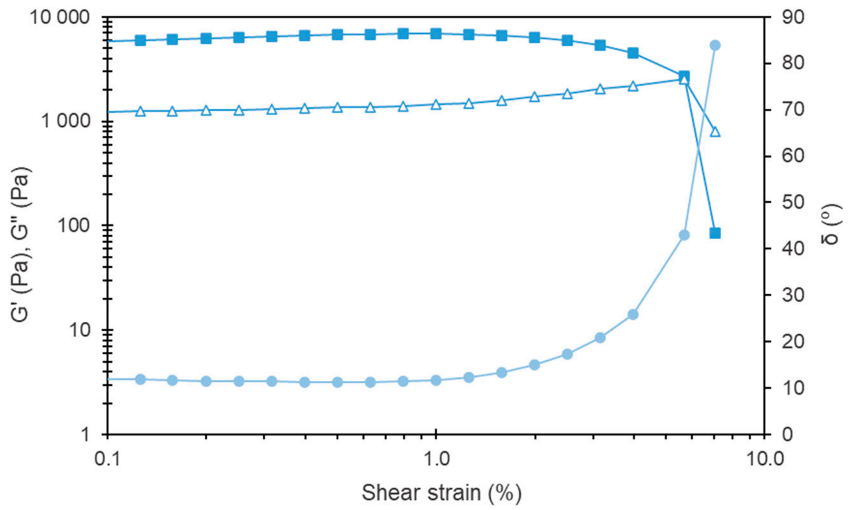

Figure S2. Amplitude sweep test at 37°C and 1 Hz. Storage modulus (G'), loss modulus (G''), and phase angle (δ) for non-sterilized blank HG (■, Δ, ●) and HG-NLC (■, Δ, ●).

The strain sweep (amplitude sweep) test was performed from 0.1% to 100% strain for blank HG and HG-NLC to define the linear viscoelastic region (LVER), where the dynamic moduli were independent of the stain applied. G' and G'' run almost parallel without a crossover point (δ = 45°) up to ~2% and ~6% strain, respectively. A strain of 0.15% was selected for subsequent sweeps to prevent network breakdown of the developed hydrogels.

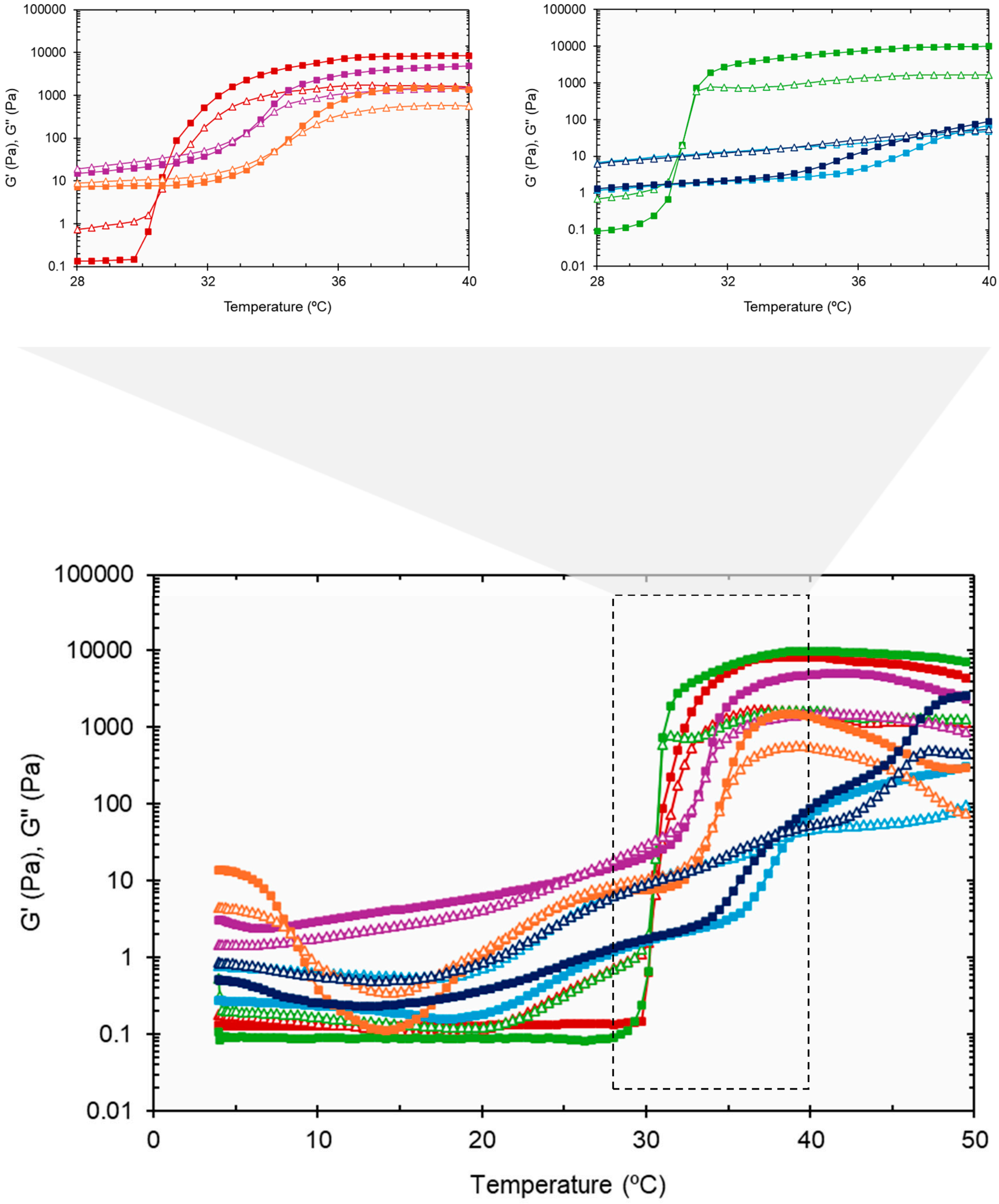

Figure S3. Storage modulus (G') and loss modulus (G'') during the temperature sweep for blank HG (■, △), blank sHG (■, △), HG-NLC (■, △), sHG-NLC (■, △), HG-NLC-DTX (■, △), and sHG-NLC-DTX (■, △).

The gelation point is determined by the G'/G'' crossover point.

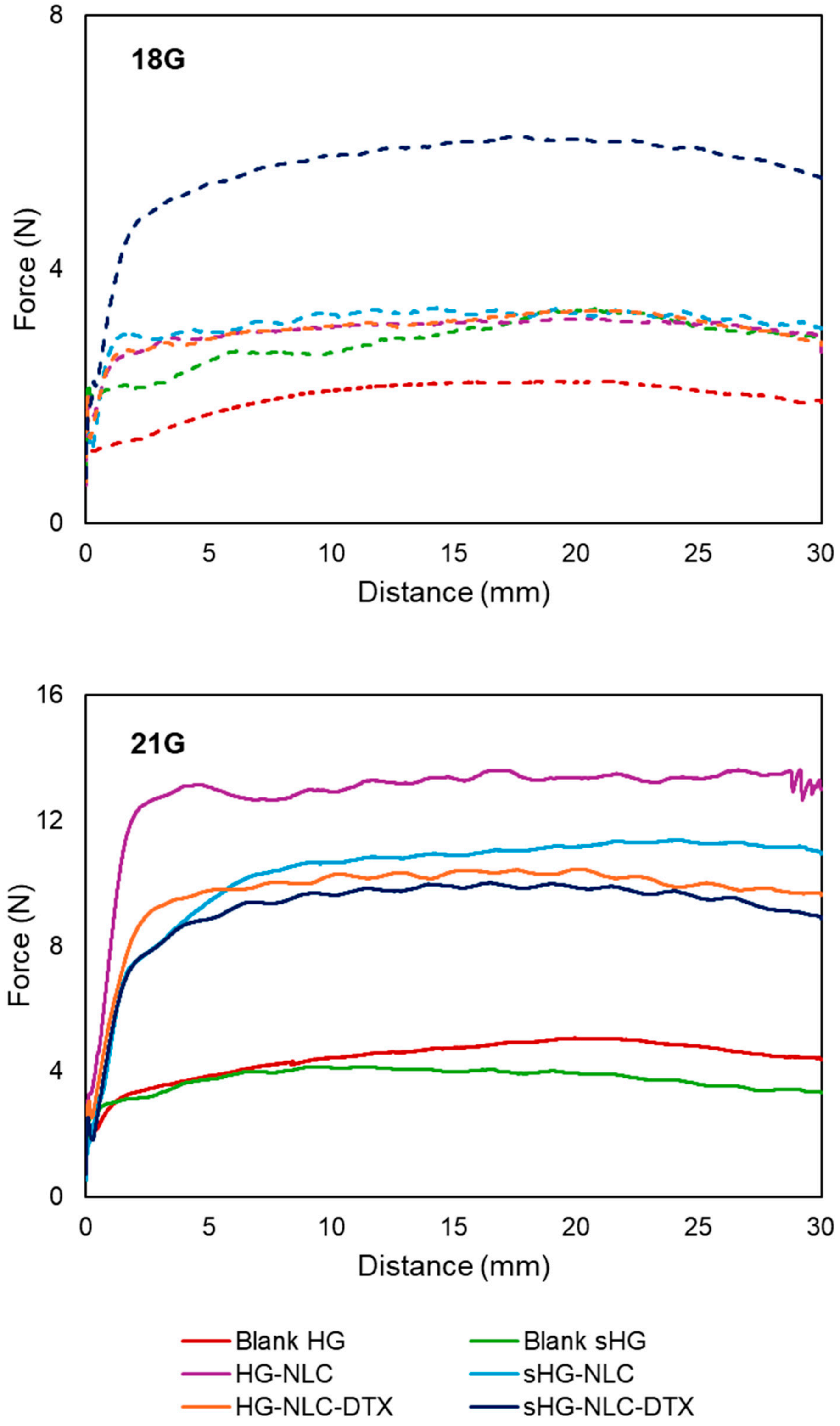

Figure S4. Force-distance profiles of the developed hydrogels in a 2.5-mL syringe extruded through 18G (**top**) and 21G (**bottom**) needles. The curves represent the average results of three injections.

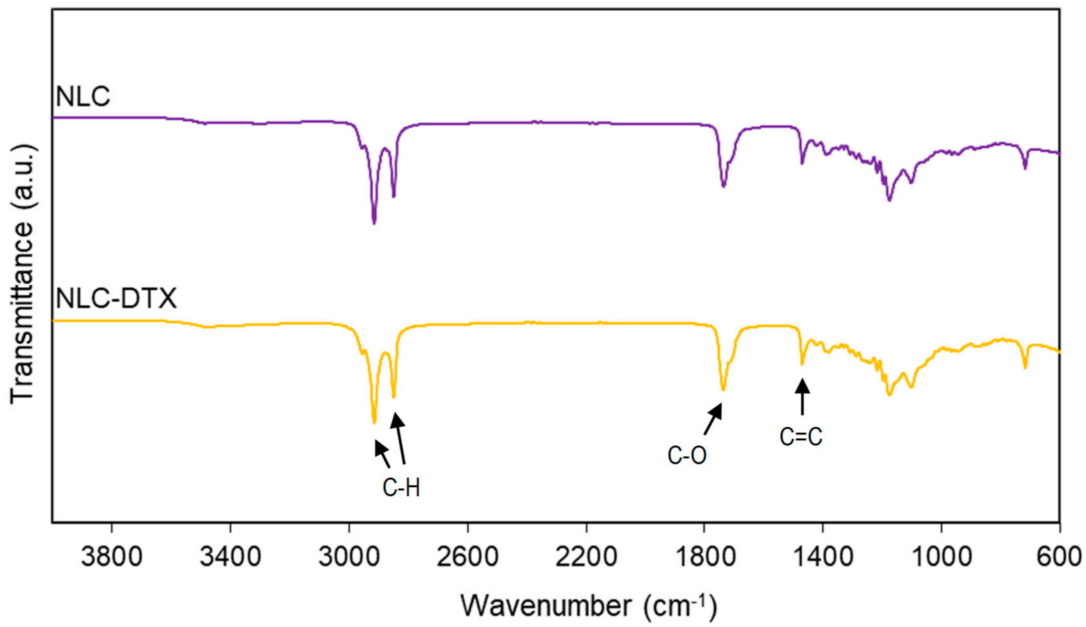

Figure S5. Fourier-transform infrared (FTIR) spectra of unloaded NLC and DTX-loaded NLC (NLC-DTX). The graphs are plotted on the same scale to allow for easy comparison.
